# Latent Tuberculosis Activation Due to Short-Term Glucocorticoid Use in Acute Systemic Lupus Erythematosus

**DOI:** 10.7759/cureus.108226

**Published:** 2026-05-04

**Authors:** Akriti Jalla, Omar Bishr, Jessica R Glover

**Affiliations:** 1 Internal Medicine-Pediatrics, University of South Florida, Tampa, USA; 2 Internal Medicine, University of Florida Health, Gainesville, USA; 3 Internal and Hospital Medicine, Moffitt Cancer Center, Tampa, USA

**Keywords:** autoimmune flare-up, glucocorticoid therapy, late-onset systemic lupus erythematosus, steroid adverse effects, steroid use, systemic lupus erythematosus (sle), tuberculosis

## Abstract

We present a case of a 57-year-old Honduran male with no past medical history who initially presented to the hospital for blurred vision, but was ultimately admitted for hypoxia and acute kidney injury. His blurred vision led to an ophthalmic examination revealing bilateral retinal detachments. The evaluation for spontaneous retinal detachment led to the discovery of a positive antinuclear antibody (ANA) and interferon gamma release assay (IGRA) for tuberculosis (TB). At the time of admission, chest radiography was negative for evidence of active TB, and all sputum testing was negative. Further workup revealed the patient met criteria for acute onset systemic lupus erythematosus (SLE). Due to worsening clinical condition, he was started on 20 mg prednisone for the treatment of acute SLE along with rifampin for latent TB. However, with ongoing clinical deterioration, repeat imaging was obtained, and it was revealed that he had conversion of latent to active TB after only 13 days of glucocorticoid exposure. Both diagnoses were later confirmed by biopsy. This case endeavors to explore the potential causes for activation of latent TB from the initiation of high-dose steroids in a time course that is weakly supported by available data, and encourages further exploration into how the combination of an autoimmune condition and immunosuppressive therapy may influence the risk for reactivation of TB.

## Introduction

The convergence of infectious diseases and rheumatology in the acute setting presents a unique challenge for clinicians. We present a case that exemplifies the diagnostic and treatment dilemmas that can occur in a patient with latent tuberculosis (TB) when presenting alongside new-onset systemic lupus erythematosus (SLE). We will discuss current guidelines for the diagnosis of latent TB in a low-prevalence setting [1], demonstrate how it intersects with the most recent diagnostic criteria for SLE [2], and discuss how we believe modifications may be necessary in the monitoring and potentially the diagnosis of latent TB when a patient is being treated with high-dose glucocorticoids for an acute presentation of SLE.

## Case presentation

**Figure 1:**
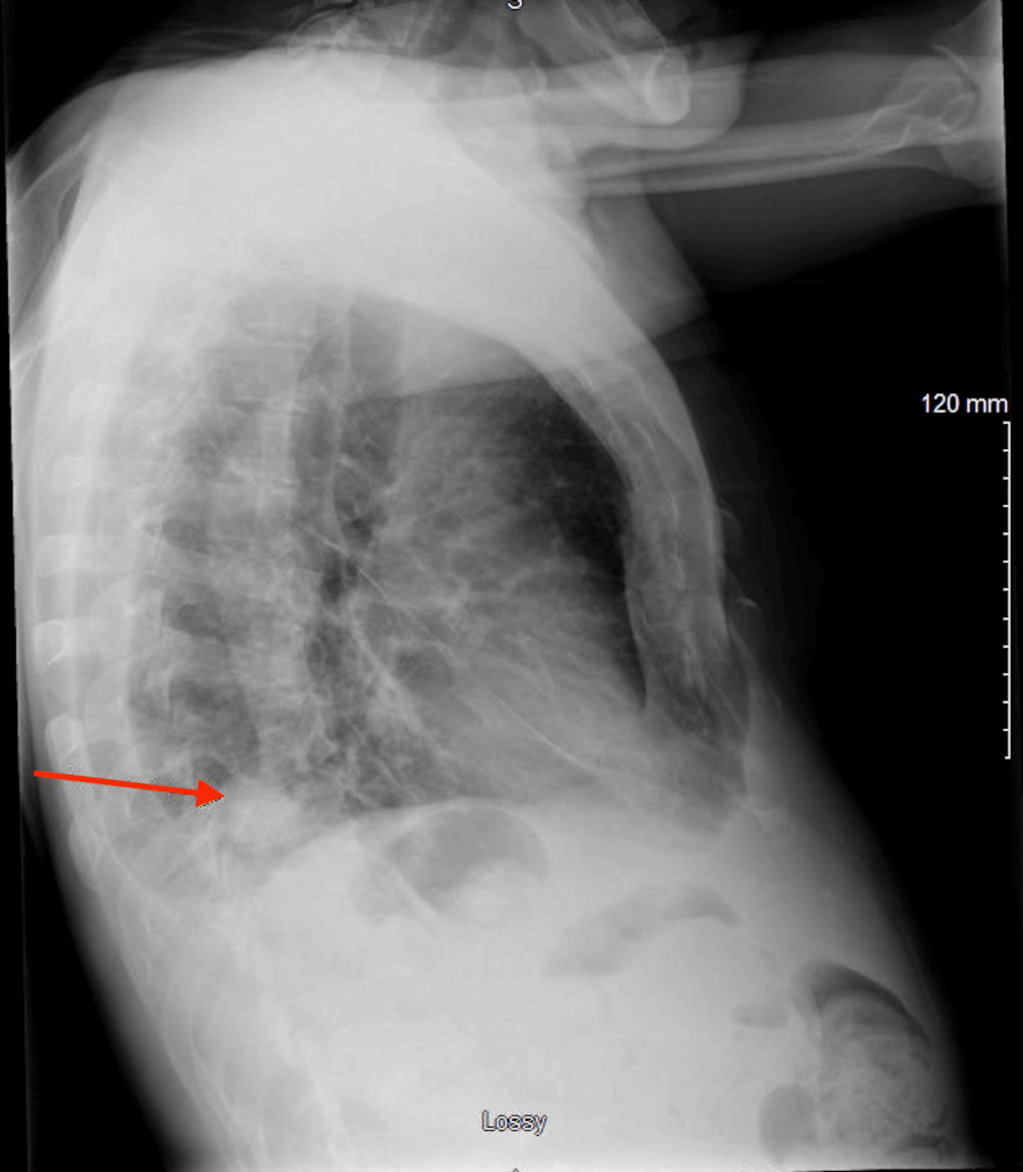
Lateral chest X-ray depicting opacity in the right lower lobe.

**Figure 2:**
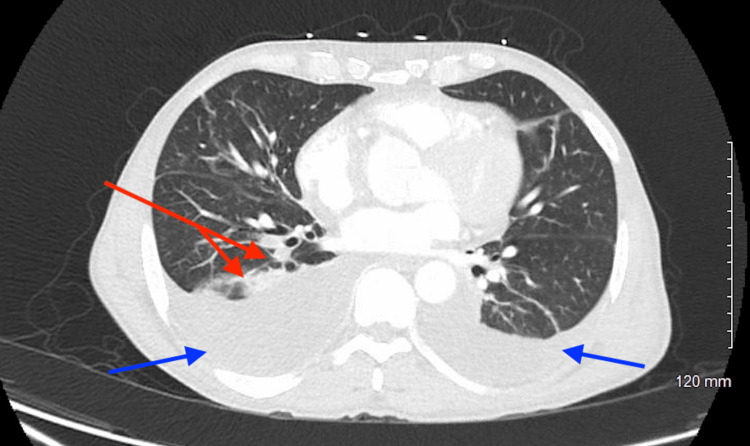
CT of the chest demonstrating right lower lobe peribronchiolar patchy opacities and bilateral pleural effusions. Red arrows = opacities; blue arrows = pleural effusions.

A 57-year-old previously healthy Honduran male was admitted with a two-week history of recurrent fevers, chills, bilateral vision changes, low back pain, flank pain, and abdominal pain. He reported being recently treated with a five-day course of levofloxacin at an outside facility for suspected community-acquired pneumonia for the same symptoms without improvement. On admission, a sepsis workup was initiated, including blood cultures and imaging studies. Initial chest X-ray revealed a round opacity projecting over the right lower lobe (Figure 1), raising concerns for pneumonia. Computed tomography (CT) imaging of the chest demonstrated patchy peribronchiolar consolidation in the right lower lung lobe (RLL) and small bilateral pleural effusions (Figure 2). Urinalysis demonstrated hematuria and proteinuria without significant pyuria. Due to the patient’s vision changes, ophthalmology was consulted on admission and diagnosed the patient with xerophthalmia and bilateral retinal detachments. The differential for this included uncontrolled type 2 diabetes mellitus, TB, syphilis, and autoimmune disease; thus, corresponding diagnostics were also ordered. Notably, the patient had emigrated from Honduras 24 years ago. He had received all immunizations, including Bacillus Calmette-Guérin (BCG), and had no known TB-positive contacts, recent travel, incarcerations, homelessness, or significant family history of autoimmune disease.

An interferon-gamma release assay (IGRA) and antinuclear antibody (ANA) were positive, along with extremely elevated erythrocyte sedimentation rate (ESR) and C-reactive protein (CRP) levels. We then measured complement components 3 and 4 (C3 and C4, respectively), which were low, consistent with the diagnostic criteria for SLE. These results are summarized in Table 1.

**Table 1:** A summary of initial screening test results for both tuberculosis and systemic lupus erythematosus.

Laboratory test	Result	Reference range
Interferon-gamma release assay	Positive	Negative
Anti-nuclear antibody	1:2560	<1:160 in males
Complement component 3	39 mg/dL	88-201 mg/dL
Complement component 4	Undetectable	14-45 mg/dL
Erythrocyte sedimentation rate	125 mm/hr	0-20 mm/hr
C-reactive protein	9 mg/dL	0-1.0 mg/dL

In accordance with the World Health Organization and the Infectious Diseases Society of America's guidelines for the rule-out of active TB, three sputum samples were also obtained at least eight hours apart [1,3]. All three samples underwent sputum *Mycobacterium tuberculosis* (MTB) polymerase chain reaction (PCR) testing and acid-fast bacilli (AFB) smears, all of which showed negative results. Importantly, although the diagnostic sensitivity of AFB sputum cultures is only 48%, when combined with the MTB PCR, whose sensitivity is 57%, the ability to rule out MTB in a low-prevalence population rises to 77%, and a second negative MTB PCR is thought to exclude nearly 100% of non-active TB cases [4]. Thus, after discussion with infectious disease consultants, the patient was removed from isolation precautions and diagnosed with latent TB infection (LTBI). Subsequent pleural fluid evaluation and lymph node biopsies over the next week were also sent for AFB smears and were negative.

During this time, the patient’s fevers and hypoxia were continuing to worsen despite broad-spectrum antibiotics and no positive blood or sputum cultures. On hospital day three, a CT angiogram of the chest and echocardiogram were completed, which led to new diagnoses of RLL pulmonary embolism, thrombosis of the left internal jugular vein, diffuse axillary and cervical lymphadenopathy, and a new small pericardial effusion. Further investigation into the patient’s positive ANA and hypocomplementemia eventually revealed positive anti-Smith, anti-dsDNA, anti-ribonucleoprotein, anti-Sjogren’s syndrome-related antigen A, and anti-histone antibodies. The anti-dsDNA antibody with the new pericardial effusion was sufficient for the patient to meet criteria for SLE in accordance with the 2019 European League Against Rheumatism (EULAR)/American College of Rheumatology (ACR) diagnostic criteria, represented in Table 2. The decision was made by rheumatology to initiate systemic corticosteroids at a dose of 20 mg prednisone daily [2]. This was done in communication with the infectious disease team, who started treatment for LTBI with rifampin on the same day.

**Table 2:** Patient-specific diagnostic criteria for systemic lupus erythematosus. EULAR: European League Against Rheumatism; ACR: American College of Rheumatology; ANA: anti-nuclear antibody. Adapted from Aringer et al. [2].

2019 EULAR/ACR criteria	Points possible	Patient’s score (based on available data on day 3)
Fever	2	2
Leukopenia	3	0
Thrombocytopenia	4	0
Autoimmune hemolysis	4	0
Delirium	2	0
Psychosis	3	0
Seizure	5	0
Non-scarring alopecia	2	0
Oral ulcers	2	0
Subacute cutaneous or discoid lupus	4	0
Acute cutaneous lupus	6	0
Pleural OR pericardial effusion	5	5
Acute pericarditis	6	0
Joint involvement	6	0
Proteinuria >0.5 g/24 h	4	0
Renal biopsy class II or V lupus nephritis	8	0
Renal biopsy class III or IV lupus nephritis	10	0
Antiphospholipid antibodies	2	0
Low C3 OR low C4	3	0
Low C3 AND low C4	4	4
Anti-dsDNA OR anti-Smith antibodies	6	6
Total (>9 + positive ANA required for diagnosis)	-	17 + positive ANA

**Figure 3:**
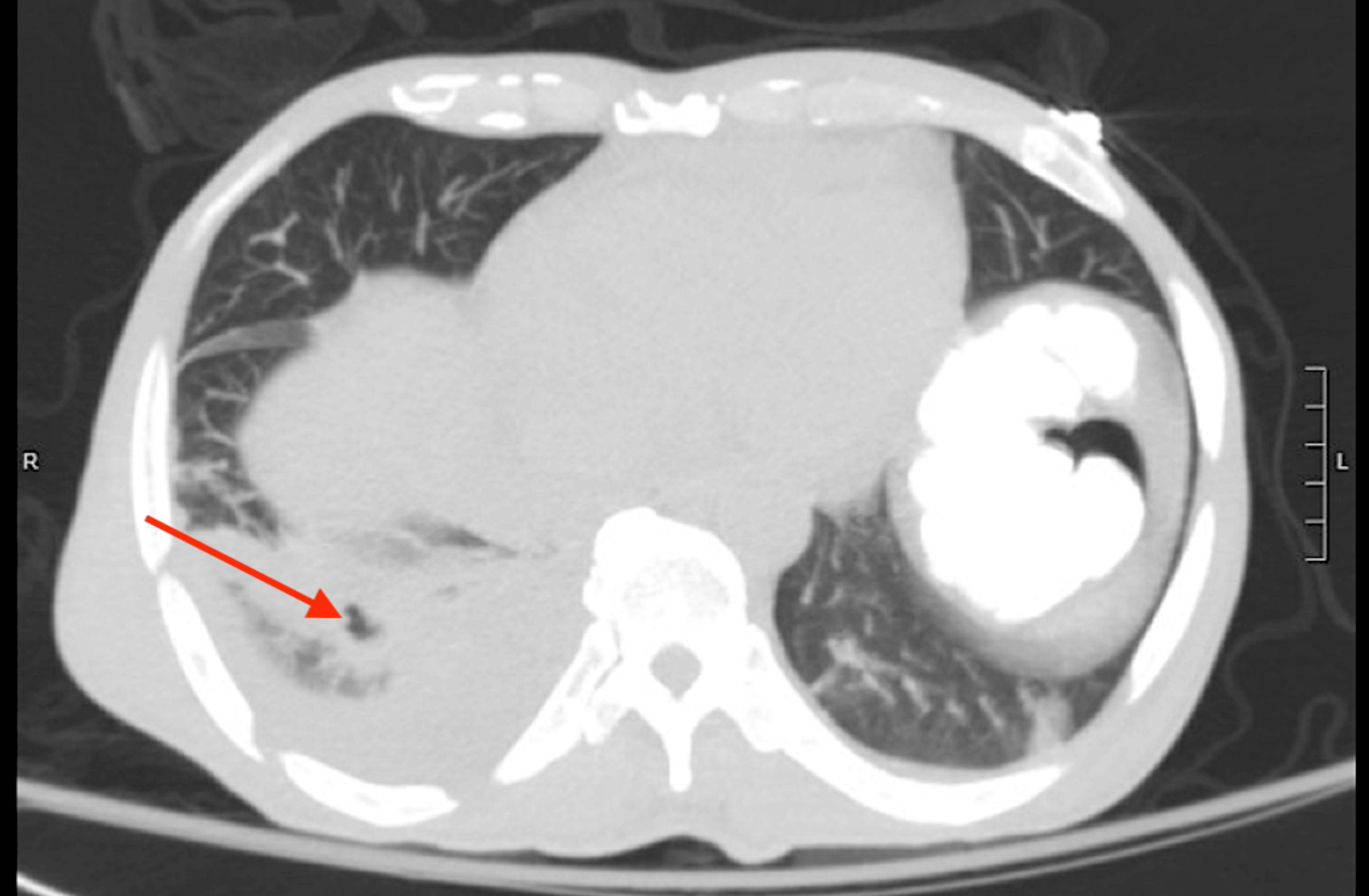
CT of the chest without contrast on hospital day 16 demonstrating new right lower lobe cavitation.

Unfortunately, the patient’s condition continued to deteriorate with worsening hypoxia from pleural effusions, which eventually required thoracentesis. As previously mentioned, the pleural fluid obtained was tested and had negative cultures, including AFB. Repeat inflammatory markers on hospital day 13, 10 days after steroid initiation, showed ESR decreased only slightly from 125 to 94 mm/hr and CRP also decreased less than expected, from 9 to 4.47 mg/dL. This caused suspicion of a secondary disease process, which came to light when the patient developed unstable atrial fibrillation three days later. Once the patient was stabilized, a CT of the chest was repeated, which demonstrated a new cavitary pulmonary lesion (Figure 3). This instantly raised concern for the transformation of latent to active TB. It also showed a worsening pericardial effusion, which was the likely cause of the new onset of atrial fibrillation. He was transitioned to rifampin, isoniazid, pyrazinamide, and ethambutol (RIPE) therapy for active TB treatment the same day by the infectious disease team. Rheumatology was reconsulted; however, they opted not to lower the patient's prednisone dose due to the unclear cause of the patient's worsening pericardial effusion. Thankfully, the patient demonstrated remarkable improvement after the transition to RIPE therapy and was discharged four days later.

Subsequent outpatient follow-up with rheumatology redemonstrated positive ANA titers, positive anti-Smith antibodies, and low complement levels, while a renal biopsy confirmed lupus nephritis. The patient was also hospitalized again shortly after discharge for worsening right pleural effusion, at which time the pleural fluid did have a positive MTB PCR, confirming active TB infection. He maintained close rheumatology and infectious disease follow-up and was successfully initiated on a biologic agent for his lupus after completing two months of RIPE and continuation therapy with isoniazid and rifampin for seven months.

## Discussion

The final diagnosis of acute late-onset SLE in a male, by itself, represents a highly atypical clinical presentation. In the last 25 years, there have been reports indicating the occurrence of late-onset SLE in patients aged 50-60 years and older, though it remains rare, accounting for only 12-18% of SLE cases [5]. Late-onset SLE, or SLE in the elderly, is distinct from the classic form of the disease. It is often a delayed or missed diagnosis because of its low incidence and atypical symptoms, with greater prevalence of serositis, cytopenias, lung involvement, and Sjogren’s syndrome than skin manifestations, photosensitivity, arthritis, and nephritis [5]. Late-onset SLE also presents with distinct lab findings, including a lower incidence of hypocomplementemia and a higher positivity rate of rheumatoid factor, anti-Smith antibody, and anti-ribonucleoprotein antibody. Despite its more benign disease course, the 10-year mortality is higher at 29%, compared with 5% for early-onset SLE. This is thought to be due to the higher burden of comorbid conditions that often present at older ages, rather than being directly attributable to SLE itself [6]. A unique aspect of this patient’s presentation relative to that of most late-onset SLE cases was his hypocomplementemia. We believe that the combination of this immunocompromised state with the addition of reduced host defenses from the glucocorticoids contributed to the rapid reactivation of the patient’s LTBI.

The human host response to mycobacterial infection relies heavily on the classical complement pathway, which involves the cleavage of C4 to form the convertase C4bC2a, which then cleaves C3 to its constituents C3a and C3b, the ligands responsible for the opsonization of *M. tuberculosis* [7]. With low complement levels, less opsonization and thus less phagocytosis via macrophages will occur. Other immune defenses become more important in controlling any non-phagocytized bacteria, as well as controlling mycobacteria that have escaped proteolysis and reside in the macrophages in their latent state. These other defenses include cytokines, neutrophils, and lymphocytes, which typically sequester the infected macrophages into the traditional granulomas characteristic of latent TB. Steroids can have a deleterious effect on the host’s ability to combat latent TB infection by reducing the production of these pro-inflammatory cytokines, such as interferon-gamma, a critical mediator of macrophage activation, by the host's T cells [7]. Steroids further cause the degranulation of leukocytes responsible for recruiting cytokines to infected cells, thereby compounding the host’s inability to combat latent infection [8].

Due to the multiple nuances of diagnosing and treating both acute late-onset SLE and LTBI, developing the correct treatment plan that prevented a potentially fatal outcome was only achievable through close collaboration between hospital medicine, rheumatology, and infectious disease specialists. Many of the diagnostic criteria for SLE, including fever, leukopenia, arthralgias, and serositis, can be present in both disease states. Furthermore, diagnosis of LTBI may be delayed due to the higher prevalence of anergic tuberculin skin tests and equivocal findings on the IGRA due to the down-regulation of interferon-gamma that can occur if steroids have already been started for an SLE flare [7]. Though this may present a source of discomfort for clinicians, where overlap in diagnostic criteria occurs in a patient with severe systemic illness, it remains important to commit to a diagnosis to prevent delaying care. In this case, despite the new findings of pericardial effusion and diffuse lymphadenopathy, which could be concerning for acutely disseminated TB, we pursued the diagnosis for which the patient clearly met diagnostic criteria, i.e., SLE. It should be noted, however, that in starting treatment for both SLE and latent TB, we acknowledged that there was limited data on the use of corticosteroids in patients with latent TB [9].

Upon reviewing recent literature, it became clearer that even short-term steroid use may represent greater harm than once believed. One case-matched study conducted in the United Kingdom, including 497 confirmed TB cases, demonstrated an adjusted odds ratio (OR) of 4.9 (95% CI: 2.9-8.3) for developing TB with current steroid use [10]. Further analysis showed that only one prescription for steroids within six months tripled the risk for active TB (OR: 3.2, 95% CI: 1.4-7.4), and any prescription equivalent to 7.5 mg prednisone or higher in the past six months had an OR of 7.0 (95% CI: 2.9-16.8) compared to no steroid exposure. These data challenge the safety of even short-term courses of corticosteroids, such as those often employed in exacerbations of rheumatologic and pulmonary conditions in patients of unknown TB status, even in low-prevalence areas. Interestingly, the same study evaluated the use of other antirheumatic and immunosuppressive drugs in the same cohort, and the risk of developing active TB was less than one-third that of high-dose steroid use (OR: 2.0, 95% CI: 1.4-2.9). Moreover, there was only a significantly increased risk in current users, not with recent (<6 months ago) or past use.

Other unique features of the patient’s TB reactivation include the rapid timeline and the fact that this occurred while he was on rifampin. It took 13 days from the initiation of steroids to the clinical deterioration that led to the diagnosis and treatment of active TB. There are only case studies supporting the observed timeline, including Patil et al.’s description of a young, healthy Indian male treated with high-dose steroids for anaphylaxis for four days who presented again two weeks later with active TB, and Pozdnyakov et al.’s case of a 64-year-old Canadian male who received five days of steroids for severe COVID-19 infection and then developed active TB [11,12]. This may be because, in practice, only 5% of patients with known latent TB treated with steroids go on to develop active TB, and this risk traditionally correlates with the dose and duration of steroid exposure. More recent data have shown that the risk of TB activation may also depend on the initial indication for steroid use. For example, there has been a preponderance of active TB cases reported in patients with a COVID-19 diagnosis who typically received 10 or fewer days of high-dose steroids [13].

Lastly, a literature review evaluating the potential risk factors for TB reactivation in SLE also concluded that steroid use is likely an independent risk factor for TB reactivation; however, the data points to the potential that during acute presentations of SLE, the up-regulation in inflammatory cytokines may effectively mask TB reactivation, and only after the initiation of glucocorticoids is it then unmasked [14]. Therefore, more research is necessary to assess if current methods of assessing for TB reactivation are sufficient when the presentation is concurrent with new-onset SLE or a disease flare, such as in the case presented here.

## Conclusions

In summary, this case report exemplifies the complex nature of diagnosing and treating acute rheumatologic illness in a patient with a latent TB infection. It sheds additional light on the possibility of an up-regulated immune response contributing to the delayed identification of active TB infection, which may then rapidly present itself upon the initiation of glucocorticoids due to the acute suppression of interferon-gamma production by helper T cells and reduced recruitment of cytokines to infected cells. It also emphasizes the critical role of interdisciplinary collaboration in optimizing patient care and the need for healthcare providers to remain vigilant against anchoring bias in the face of evolving clinical presentations. This case also serves as a data point for future investigators and research into how to improve our risk stratification of individuals diagnosed with latent TB who have comorbid conditions that may require immunosuppressive therapies. Additional research is also necessary to determine if the current methods recommended to diagnose active TB infection are equally valid during periods of acute illness that is not attributed to TB due to the potential masking of active infection by the up-regulated immune response to a non-TB illness.
